# Opportunistic Pathogens and Elements of the Resistome that Are Common in Bottled Mineral Water Support the Need for Continuous Surveillance

**DOI:** 10.1371/journal.pone.0121284

**Published:** 2015-03-24

**Authors:** Maria Fernanda Falcone-Dias, Daniela Centrón, Fernando Pavan, Adriana Candido da Silva Moura, Felipe Gomes Naveca, Victor Costa de Souza, Adalberto Farache Filho, Clarice Queico Fujimura Leite

**Affiliations:** 1 School of Pharmaceutical Sciences, UNESP- Univ Estadual Paulista, Araraquara, SP, Brazil; 2 Departamento de Microbiología, Facultad de Medicina, UBA, Buenos Aires, Argentina; 3 Instituto Leônidas e Maria Deane—ILMD, FIOCRUZ, Manaus, AM, Brazil; NERC Centre for Ecology & Hydrology, UNITED KINGDOM

## Abstract

Several differences concerning bacterial species, opportunistic pathogens, elements of the resistome as well as variations concerning the CFU/mL counts were identified in some of the five most marketed bottled mineral water from Araraquara city, São Paulo, Brazil. Two out of five brands tested were confirmed as potential source of opportunistic pathogens, including *Mycobacterium gordonae*, *Ralstonia picketti* and *Burkholderia cepacia* complex (Bcc). A total of one hundred and six isolates were recovered from four of these bottled mineral water brands. Betaproteobacteria was predominant followed by Alphaproteobacteria, Gammaproteobacteria and Firmicutes. Ninety percent of the bacteria isolated demonstrated resistance to seventeen of the nineteen antimicrobials tested. These antimicrobials included eight different classes, including 3rd and 4th generation cephalosporins, carbapenems and fluoroquinolones. Multidrug resistant bacteria were detected for fifty-nine percent of isolates in three water brands at counts up to 10^3^ CFU/ml. Of major concern, the two bottled mineral water harboring opportunistic pathogens were also source of elements of the resistome that could be directly transferred to humans. All these differences found among brands highlight the need for continuous bacteriological surveillance of bottled mineral water.

## INTRODUCTION

Sales of mineral water have been increasing around the world, possibly because of the decreased safety of some tap water for consumption [1]. Spring waters typically contain a characteristic natural bacterial flora [2], [3]. However, natural mineral water may not be subjected to any disinfection treatment [4]. Thus, bottled mineral waters are not sterile environments, but they are complex ecosystems with a high phenotypic and genetic diversity of autochthonous bacteria [3]. According to numerous studies, the microorganisms most frequently found in bottled natural mineral water are aerobic heterotrophs belonging primarily to the Alpha-, Beta- and Gammaproteobacteria [5], [6], [7], [8]. Some studies have also found bacteria that were resistant to various antibiotics in bottled mineral water [6], [8].

Bacteria that live in natural habitats are potential sources of antibiotic resistance genes that can be transmitted to human commensal and pathogenic bacteria [9], [10], [11]. Evidence shows that at least some clinically relevant resistance genes have originated in environmental bacteria [12]. An important part of the dispersal and evolution of antibiotic resistance depends on aquatic environments, because bacteria from different origins (human, animal and environmental) are able to mix, and resistance evolves as a consequence of the exchange of genes, genetic platforms, and genetic vectors. Therefore, this water provides a way of disseminating antibiotic resistance among environmental, human and animal populations through drinking water [13]. Another preoccupation with drinking water is the presence of nontuberculous mycobacteria (NTM) and other opportunistic pathogens, without evidence of person-to-person transmission, it is proposed that humans, are infected from environmental sources [14], [15], [16].

Therefore, the presence of opportunistic pathogens and antibiotic resistant bacteria in mineral water can pose a serious public health problem. At present, there is no literature on this subject in Brazil, and thus the aim of this study was to investigate the quality of the bottled mineral water focusing on the bacterial diversity and resistance phenotypes of the 5 most consumed brands that are marketed in Araraquara city, São Paulo, Brazil.

## MATERIALS AND METHODS

### 1. Sampling

Five brands (named A, B, C, D and E) of noncarbonated natural mineral water were analyzed. Two bottles of each brand were mixed in a sterile glass bottle to complete the volume needed for all analyzes (total cultivable bacteria and mycobacteria).These waters were contained in 1.5 liter polyethylene terephthalate (PET) bottles and were purchased from a retail outlet in Araraquara city, São Paulo, Brazil, but came from sources in two different regions of Brazil. After purchased, the mineral water bottles were stored at room temperature (25 to 27°C) prior to investigation for a maximum of 24 hours.

### 2. Total cultivable and antibiotic-tolerant bacteria

#### 2.1. The enumeration of total cultivable and antibiotic-tolerant bacteria.

Two culture media widely used for the microbiological quality control of drinking water were employed: R2A (Himedia, Mumbai, India) and *Pseudomonas* isolation agar (PIA; Himedia, Mumbai, India). The respective antimicrobial-tolerant subpopulations were enumerated on the same media supplemented with 32 mg/L amoxicillin or 4 mg/L ciprofloxacin. Bacteriological analyses were performed by using the membrane filtration method [8]. Volumes of 1, 10 and 100 ml (for culture medium with antibiotic) and 1, 10 ml and decimal dilutions (-2 and -1) (for culture medium without antibiotic) of water samples were filtered through cellulose ester membranes (0.45 μm pore size, 47 mm diameter, Millipore), which were placed onto the different culture media described above and incubated for 48 h at 30°C. All analyses were made in triplicate. After the incubation period, the number of colony forming units (CFU) was registered on the basis of filtering membranes containing between 10 and 100 colonies. The CFU/ml values were registered for each culture medium.

#### 2.2. Bacterial isolation and preliminary characterization.

Bacteria were isolated after the visual examination of triplicate culture plates, which exhibited a countable number of CFUs until 48 h of incubation. Culture plates on which no growth was observed within this period were incubated until 7 days. During the incubation period, the plates were examined and bacteria were isolated and purified. Representatives of all colony types were selected for further purification, according to the criterion described by [8]; all colonies of morphotypes represented by up to two colonies, 50–75% colonies of morphotypes represented between three and nine colonies, and five colonies with morphotypes represented by more than nine colonies. The cultures were purified by sub-culturing on R2A (Himedia, Mumbai, India). Pure cultures were preserved at -80°C in Luria Bertani broth (Acumedia, Lansing, USA) supplemented with 15% (v/v) glycerol.

#### 2.3. Bacterial identification and typing.

The bacterial isolates were genotyped by random amplified polymorphic DNA (RAPD) analysis, using the primer M13, to detect possible clones (the same bacterium isolated on the same filtering membrane) [8]. The RAPD genotypes were analyzed visually and whenever two or more isolates from the same filtering membrane presented an identical profile, they were considered to be clones. All non-clonal bacterial isolates were identified by analyzing the 16S rRNA gene sequence using the primers 27F and 1492R. All non-clonal bacterial amplicons were purified as described by [17], [18] with modifications by [19]. The sequencing reactions were performed with BigDye terminator v3.1 and approximately 10–40 ng of each purified amplicon with 27F and 1492R primers. Capillary electrophoresis was performed using POP-7 and an ABI 3130 DNA sequencer, followed by an initial evaluation with the Sequencing Analysis software (Applied Biosystems, v5.3.1). Only those nucleotide sequences with a phred score cut-off of ≥ 20 (higher than 99% of accuracy) were used for contig assembly. The nucleotide sequences of approximately 1300 bp were used to query the GenBank library (Accession numbers KP744123- KP744146) to arrive at the closest type strain and thus attain a species affiliation and possible identification to that level. To compare the 16S rRNA gene sequences of isolates from different samples, the nucleotide sequences were aligned with ClustalW from MEGA software, version 5.1, and dendrograms were created by using the neighbor-joining method based on a model by Jukes and Cantor.

#### 2.4. Determining antibiotic resistance phenotypes.

Antibiotic resistance phenotypes were determined by agar diffusion method [20].The bacterial inoculum was prepared by bacterial dilution in sterile MilliQ water. This inoculum was seeded in plates with Muller Hinton agar (Oxoid, Basingstoke, England) and after placed the antibiotics disks. The tested antibiotics were as follows: the beta-lactams ticarcillin (75 μg), ticarcillin/clavulanic acid (75/10 μg), ceftazidime (10 μg), cefepime (30 μg), imipenem (10 μg), andmeropenem (10 μg); the aminoglycosides streptomycin (10 μg) and gentamicin (10 μg); tobramycin (10 μg) andamikacin (30 μg); the quinolones levofloxacin (5 μg), nalidixic acid (30 μg), and ciprofloxacin (10 μg); the polymyxincolistin sulfate (10 μg); the sulfonamide sulfamethoxazole (25 μg) andsulfamethoxazole/trimethoprim (23.75/1.25 μg); fosfomycin (50 μg); rifampicin (5 μg); and tetracycline (30 μg) (Oxoid, Basingstoke, England). The strains *Escherichia coli* ATCC 25922 and *Pseudomonas aeruginosa* DSM 1117 (ATCC 27853) were included as quality controls. Phenotypes were classified as resistant, intermediary or susceptible according to the manufacturer’s instructions.

### 3. Mycobacteria

#### 3.1. The isolation and preliminary characterization of mycobacteria.

A 1000 ml volume of each brand was filtered through cellulose ester membranes (0.45 μm pore size, 47 mm diameter, Millipore), and the membranes were macerated with phosphate buffered saline for 5 minutes. The biomass that was concentrated on the membrane was treated with 5 ml of 4.0% sulfuric acid solution for 10 minutes and then neutralized with sodium hydroxide solution until reaching a neutral pH (pH = 7.0). 0.2 ml of this material was seeded into four tubes with Middlebrook 7H10 (Difco, Sparks, USA) supplemented with OADC (oleic acid, albumin, dextrose and catalase) (Sigma-Aldrich).Two tubes were incubated at 30°C and the others two were incubated at 37°C. The incubating tubes were checked weekly for the presence or absence of colony formation. Suspected colonies were confirmed as acid-fast bacilli (AFB) by using the Ziehl-Neelsen technique. Reseeding was then performed in 7H10, which was incubated at the original isolation temperatures (30° or 37°C) for the subsequent identification of the species.

#### 3.2. Identifying mycobacteria.

For the extraction of DNA a loopful of mycobacteria grown on 7H10 medium was suspended in 300 mL of TE buffer (10 mMTris, 1 mM EDTA pH 8.5) and subjected to 3 cycles of boiling (10 min. at 100°C) and freezing (20 minutes at -20°C). The PRA technique (PCR-Restriction Enzyme Analysis) was used to identify the mycobacterial isolates. A 439 bp fragment was amplified from the hsp65 gene for PCR. The DNA sequence of the PCR product was then digesting with the enzymes BstEII (Fermentas, Glen Burnie, MD, USA) and HaeIII (Fermentas, Glen Burnie, MD, USA) according to [21]. The products of the digestion reaction was separated and visualized by gel electrophoresis. The pattern of bands obtained was then compared with the banding patterns obtained from reference strains according to the database PRASITE (http://app.chuv.ch/prasite).

### 4. Statistical analysis

Data on the log (CFU per 100 milliliter) for each water brand were compared among the culture media with antibiotics and the same culture media without antibiotics, and data on the log within each culture medium were compared among the three brands by using an analysis of variance and a post hoc Tukey's test (SPSS 17.0 for Windows), at a significance level (P) of 0.05.

## RESULTS

### The total cultivable bacteria found in each brand differs in taxa and abundance

The CFU counts for the 5 brands of mineral water were performed at 48 h as described in Materials and Methods (Table 1). The culture plates on which no growth was observed within this period were re-incubated for more 5 days only for isolation of strains with low level of growth. As a whole, significant differences in the CFU/mL count were observed between the brands (Table 1). In the absence of antibiotics, the cultivable bacteria ranged from 4.29–6.49 Log CFU/100 ml until 48 h. For all brands tested, the CFU/ml values were significantly higher on R2A and on PIA plates without antibiotics than on the same medium supplemented with amoxicillin or ciprofloxacin (P<0.05). In the presence of amoxicillin, the average values for cultivable bacteria counts ranged from 0.68–4.72 Log CFU/100 ml. The addition of ciprofloxacin to the culture medium caused a more pronounced inhibitory effect on bacterial growth than amoxicillin, with the exception of the brand A. In brand E, growth was only observed on medium supplemented with ciprofloxacin after a longer period of incubation (3 to 7 days) (Table 1).

**Table 1:** Colony forming units (CFU) of culturable bacteria per 100 ml of bottled mineral water, as recovered on R2A and PIA and in the same culture media supplemented with amoxicillin (AML, 32 mg/L) or ciprofloxacin (CIP, 4 mg/L) at 48 h of incubation.

Brand	Mean ± SD* (Log CFU/100 ml)											
	R2A		R2A-AML		R2A-CIP		PIA		PIA-AML		PIA-CIP	
**A**	4.70 ± 0.09	^a, 1^	1.38 ± 0.05	^b, 1^	1.47 ± 0.06	^b, 1^	4.29 ± 0.10	^a, 1^	0.68 ± 0.35	^b, 1^	0.67 ± 0.19	^b, 1^
**B**	5.34 ± 0.03	^a, 2^	4.71 ± 0.05	^b, 2^	1.85 ± 0.00	^c, 2^	4.42 ± 0.15	^a, 1^	3.11 ± 0.11	^b, 2^	2.19 ± 0.34	^c, 2^
**C**	< 0	^3^	< 0	^3^	< 0	^3^	< 0	^2^	< 0	^3^	< 0	^3^
**D**	< 0	^3^	< 0	^3^	< 0	^3^	< 0	^2^	< 0	^3^	< 0	^3^
**E**	6.49 ± 0.14	^a, 4^	4.72 ± 0.05	^b, 2^	< 0	^c, 3^	5.79 ± 0.02	^a, 3^	3.05 ± 0.10	^b, 2^	< 0	^c, 3^

Letters indicate significant (P<0.05) differences between culture medium with antibiotic and the same culture medium without antibiotic for each brand;

Numbers indicate significant differences (P<0.05) between the brands for each culture medium.

**Fig 1:**
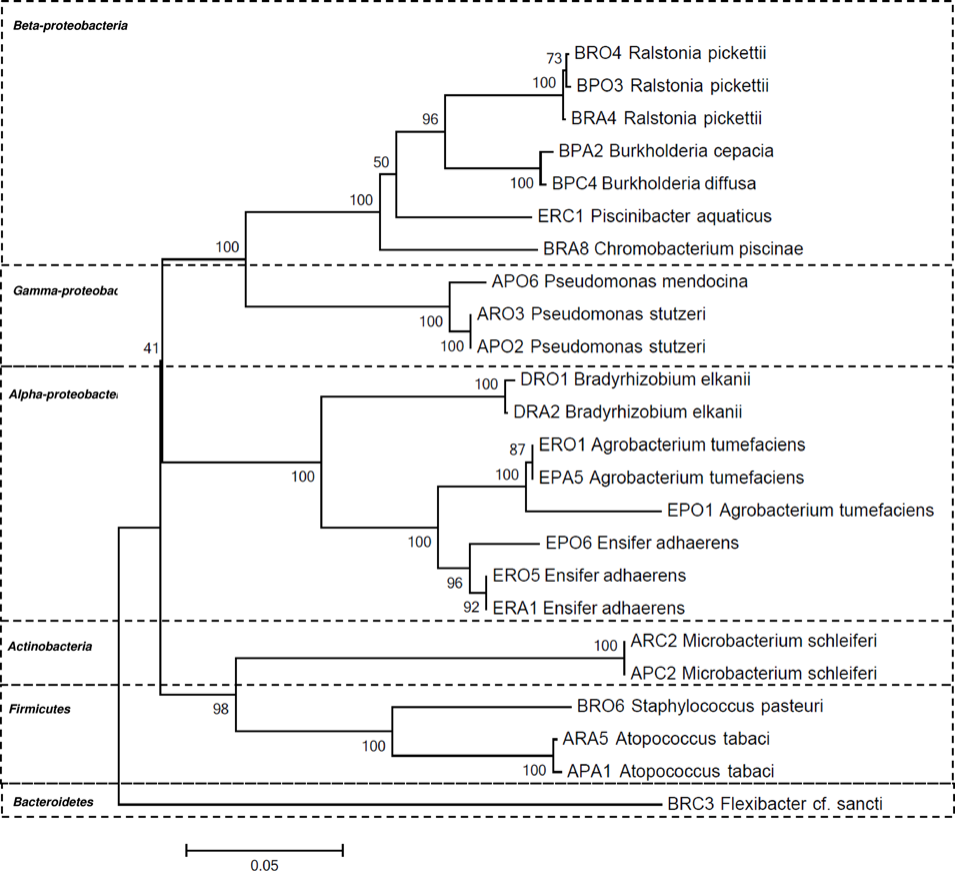
A dendrogram representing the diversity of species isolated from mineral waters. The isolate designation are of the IMAi type, in which I is the water brand (A, B, D or E); M, the culture medium on which it was isolated (R, for R2A or P, for PIA); A, indicates the supplementation with antibiotic (O, none, A, amoxicillin, and C, ciprofloxacin); and i indicates the number of isolates that were used for 16S rRNA gene sequence analysis.

*SD, standard deviation; values are the means of three determinations;

In total, 106 bacterial isolates were recovered during this study, including 8 isolates from Band D which only grew after 7 days of incubation (Fig. 1). From brand A, we collected 36 isolates (10 on R2A, 5 on R2A-amoxicillin, 5 on R2A-ciprofloxacin, 7 on PIA, 4 on PIA-amoxicillin and 5 on PIA-ciprofloxacin); for brand B we also recovered 36 isolates (8 on R2A, 8 on R2A-amoxicillin, 5 on R2A-ciprofloxacin, 5 on PIA, 5 on PIA-amoxicillin and 5 on PIA-ciprofloxacin); from brand D we recovered 8 isolates (5 on R2A and 3 on R2A-amoxicillin); and from brand E we recovered 26 isolates (5 on R2A, 5 on R2A-amoxicillin, 3 on R2A-ciprofloxacin, 8 on PIA and 5 on PIA-amoxicillin). All these isolates were submitted to RAPD analysis; when two or more isolates from the same filtering membrane presented identical RAPD profiles, they were considered as clones. Then, every non-clonal bacterial isolates were identified on the basis of their 16S rRNA gene sequence analysis. These isolates showed similar values from at least 95% to 100% with type strains of validly named species in the GenBank database, within the phyla Firmicutes, Actinobacteria, Bacteroidetes or Proteobacteria (Alpha-, Beta- and Gamma-divisions). Upon using 16S rRNA-based analysis and making a bioinformatics analysis with the GenBank database, the 106 isolates were affiliated with 14 species with 4 in brand A, 6 in brand B, 1 in brand D and 3 in brand E (Table 2, Fig. 1). Interestingly, the abundance and pattern of taxa differed for each water brand. Isolates affiliated with the species *Pseudomonas stutzeri* and *Microbacterium schleiferi* predominated in brand A (Table 2). In brand B predominated the isolates affiliated with the species *Ralstonia picketti*. *Bradyrhizobium elkanii* was the only species recovered from brand D. In brand E, the predominant isolates were affiliated with the species *Agrobacterium tumefaciens* and *Ensifer adhaerens*. Considering the whole set of isolates, the species affiliated with Proteobacteria prevailed.

**Table 2:** Antibiotic resistance patterns observed for each species, corresponding to a total of 106 bacterial isolates from mineral waters.

Brand	Species	Tax.^a^	N° isolates	Antibiotics^c^																			MDR^b^
				Beta-lactams						Aminoglycosides				Quinolones			Polymyxin	Sulfonamides				Tetracycline	(n° ofresistant classes/
				1	2	3	4	5	6	7	8	9	10	11	12	13	14	15	16	17	18	19	n°of classes tested)
**A**	*Pseudomonas stutzeri*	GP	16	S	S	S	S	S	S	S	S	S	S	S	S	S	S	S	S	**R**	**R**	S	-
	*Pseudomonas mendocina*	GP	1	S	S	S	S	S	S	S	S	S	S	S	S	S	S	S	S	**R**	**R**	S	-
	*Atopococcus tabaci*	F	9	S	S	S	**R**	S	S	S	**R**	S	S	**R**	**R**	S	S	S	S	**R**	S	S	MDR (4/8)
	*Microbacterium schleiferi*	A	10	S	S	S	**R**	S	S	S	**R**	S	S	**R**	**R**	**R**	S	S	S	**R**	S	S	MDR (4/8)
**B**	*Ralstonia picketti*	BP	17	**R**	**R**	S	**R**	S	**R**	**R**	S	**R**	**R**	I	**R**	S	**R**	S	S	**R**	**R**	S	MDR (6/8)
	*Staphylococcus pasteuri*	F	1	S	S	S	S	S	S	S	S	S	**R**	S	S	S	S	S	S	S	S	S	-
	*Chromobacterium piscinae*	BP	3	S	S	S	S	S	S	S	S	S	S	S	S	S	S	S	S	S	S	S	-
	*Burkholderia cepacia*	BP	5	**R**	**R**	S	S	S	S	**R**	**R**	**R**	**R**	I	**R**	**R**	**R**	**R**	S	**R**	**R**	**R**	MDR (8/8)
	*Flexibacter cf*. *Sancti*	B	5	**R**	S	**R**	**R**	S	S	**R**	**R**	**R**	**R**	**R**	**R**	**R**	**R**	S	S	**R**	**R**	S	MDR (6/8)
	*Burkholderia diffusa*	BP	5	**R**	**R**	S	S	S	S	**R**	**R**	S	**R**	S	**R**	I	**R**	**R**	S	**R**	**R**	**R**	MDR (8/8)
**E**	*Agrobacterium tumefaciens*	AP	12	S	S	S	S	S	S	S	S	S	S	S	S	S	S	S	S	**R**	S	S	-
	*Ensifer adhaerens*	AP	11	S	S	S	S	S	S	S	S	S	**R**	**R**	S	S	S	S	S	**R**	S	S	MDR (3/8)
	*Piscinibacter aquaticus*	BP	3	S	S	S	S	S	S	S	S	S	S	**R**	S	S	**R**	S	S	S	S	S	-

It was not possible to perform a disc diffusion assay onthe *Bradyrhizobiumelkanii* strain that was recovered from brand D because the bacteria only showed growth after 7 days.

R, resistant; S, sensible; I, intermediate

^a^Taxonomical identification: AP, Alphaproteobacteria; BP, Betaproteobacteria; GP, Gammaproteobacteria; B, Bacteroidetes; A, Actinobacteria; and F, Firmicutes.

^b^MDR (multiple antibiotic resistant), represents species resistant to 3 or more distinct antibiotic classes.

^c^Antibiotics: 1, ticarcillin (75 μg); 2, ticarcillin-clavulanic acid (75/10 μg); 3, cefepime (30 μg); 4, ceftazidime (10 μg); 5, imipenem (10 μg); 6, meropenem (10 μg); 7, gentamicin (10 μg); 8, tobramycin (10 μg); 9, amikacin (30 μg); 10, streptomycin (10 μg); 11, nalidixic acid (30 μg); 12 ciprofloxacin (10 μg); 13 levofloxacin (5 μg); 14, colistin (10 μg); 15, sulfamethoxazole (25 μg); 16, sulfamethoxazole/trimethoprim (1.25 μg trimethoprim and 23.75 μg sulfamethoxazole); 17, fosfomycin (50 μg); 18, rifampicin (5 μg); and 19, tetracycline (30 μg).

### Elements of the resistome vary by brand

**Fig 2:**
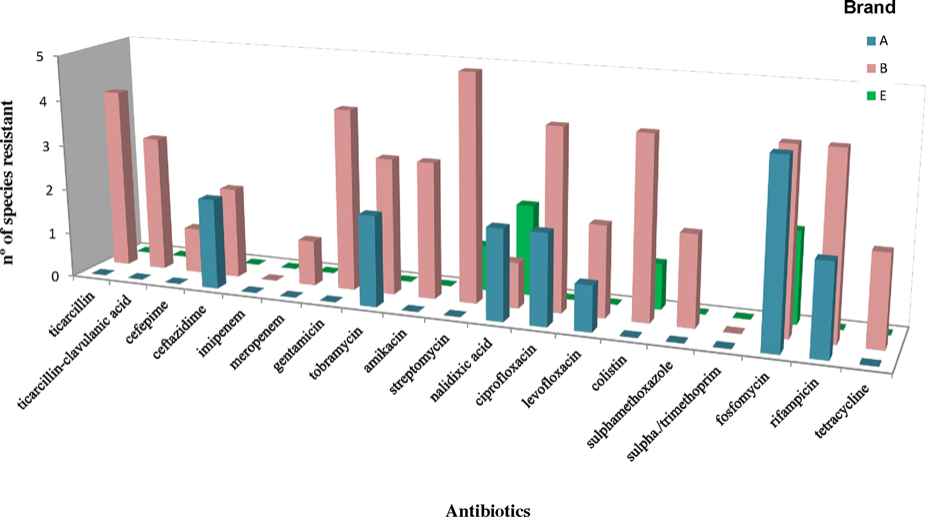
The resistome of three mineral water brands.

The resistome is the collection of genes that could contribute to an antibiotic resistance phenotype [9]. While fosfomycin was the antibiotic that showed the higher number of resistant species (n = 10), resistance to imipenem and to sulfamethoxazole/trimethoprim were not observed among the 106 isolates (Table 2, Fig. 2). In our study, we found that all three brands (A, B, and E) possessed at least one isolate that was resistant to three or more classes of antibiotics, and thus, these isolates were considered to be multidrug resistant (MDR, Table 2). Two MDR phenotypes were observed in brand A, four MDR in brand B and one in brand C (Table 2). Resistance patterns were classified as MDR(n), with n indicating the number of different antibiotic classes to which resistance was observed. Patterns MDR(3) to MDR(8) were observed, with brand B exhibiting the highest frequency of MDR (6 and 8) phenotypes. The species in the *Burkholderia* genus exhibited the highest number of MDR(8) phenotype patterns (Table 2). Isolates of the same species (100% 16S rRNA gene sequence similarity) with different RAPD profiles that were found in the same brand showed identical antibiotic resistance phenotypes (data not shown). This finding suggests that the resistance phenotype is fairly stable for each species in a brand. Each brand showed that specific elements of the resistome were supported by particular antibiotype patterns (Fig. 2).

### Opportunistic bacteria in mineral water

Several opportunistic bacteria were identified in isolates from brands A and B. The PRA technique was used to identify the mycobacterial isolate through the hsp65 gene amplification and analysis of restriction fragment polymorphisms after digesting with the enzymes *Bst*EII and *Hae*III. Analyses of restriction patterns and strain determinations were made according to the database at PRASITE. Thereby the *Mycobacteria* spp. strain recovered from brand A in the 7H10 medium incubated at 37°C for 14 days was identified as *Mycobacterium gordonae*. In brand A, we also found *P*. *stutzeri* and *P*. *mendocina* while in brand B we identified *Burkholderia difusa*, *Ralstonia picketti*, and the Bcc.

## DISCUSSION

A wide diversity of bacterial species was found in four of the five most consumed mineral water brands from a city of Brazil which were chosen for this analysis (Table 2, Fig. 1). Also, each brand evidenced particular bacterial species not shared by other brand. This is in agreement with previous studies that showed that the characteristics of the water source influence the diversity of the bacterial population in a mineral water [3], [8], [22]. In the current study, we found the predominance of Betaproteobacteria followed by Alphaproteobacteria, Gammaproteobacteria and Firmicutes (Table 2, Fig. 1). Other studies also found the predominance of Beta- and Alphaproteobacteria in bottled mineral waters from Germany, and Norway and Portugal, respectively, although the species are different [5], [7], [8]. In the current study, we also found opportunistic pathogens capable of causing infections in vulnerable individuals such as Bcc, *P*. *stutzeri*, *P*. *mendocina* and *M*. *gordonae*. *Ralstonia picketti*, Bcc, *P*. *stutzeri* and *P*. *mendocina* can infect and cause lung decline in cystic fibrosis patients. Disturbingly, both Bcc and *Ralstonia picketti* which are most of the relevant pathogens for cystic fibrosis patients [23] were found in the same brand namely brand B. Not only some genetic species of the Bcc can be very virulent among cystic fibrosis patients, but also it has been shown that they are transmitted from the environment to this community of patients [24], [25]. One previous study also identified Bcc in mineral water from Italy (6). Both findings showed the need of surveillance of this complex in bottled mineral water that is for consumption of cystic fibrosis patients, and suggest that the identification of this complex could also assist in the determination of the sanitary condition of bottled mineral waters. On the other hand, nontuberculous mycobacteria (NTM), including *M*. *gordonae*, can affect immunocompromised patients under some circumstances. Although there are numerous reports describing the presence of NTM in drinking water, in bottled table water and also in mineral water [26], [27], [28], [29], the presence of NTM identified as *M*. *gordonae* was observed in only one brand of mineral water in this study. *M*. *gordonae* is rarely considered to be clinically significant, although some infections have occasionally been reported in immunocompromised patients or patients infected with the human immunodeficiency virus [30]. NTM epidemiological studies have demonstrated that hospital water systems can be a source of infection or specimen contamination, especially in the case of *M*. *gordonae* [31]. It has previously been recommended the search for NTM in bottled water as a useful index of the water's hygienic quality [27].

Significant differences were observed between the brands concerning the CFU/mL counts. This variation was expected by some factors known as the characteristics of the water source that influence the bacterial population of mineral water [3], [19]. Furthermore, variations in the heterotrophic plate count (HPC) after bottling and/or during storage may occur related to long storage times for mineral water, to the influence of the materials, to the relative contributions of attached (e.g., biofilms) to unattached microbes and the regrowth (from small initial populations) as opposed to the resuscitation of existing microbes [19], [32], [33], [34]. Although it has been shown that the HPC alone does not directly relate to health risks for the population in general [35], these bacteria members of the HPC can cause the deterioration of bottled water when present in high concentrations, in addition to alterations in the product odor and flavor [36].

Concerning the antimicrobial resistant phenotypes of the 106 isolates tested, we found that they showed resistance to 17 antibiotics belonging to eight different classes, including 3^rd^ and 4^th^ generation cephalosporins, carbapenems and fluoroquinolones. Resistant organisms in water can represent the intrinsic resistance of normal microbial populations, or they can be the result of contamination by anthropogenic sources such as runoff from agricultural areas or from the use of antibiotics in aquaculture [12]. However, it is difficult to identify the environmental bacteria that are the natural and acquired resistance phenotypes, because information on antibiotic resistance for most of the bacterial groups detected in mineral water is scarce. As a consequence, it is also difficult to estimate the contributions of bacteria from bottled mineral water to enrich the human antibiotic resistome [8].

The detection of multidrug resistant (MDR) bacteria in mineral water deserves more attention when it has densities as high as 10^3^ CFU/mL. The bacterial isolates with the highest MDR indices were Betaproteobacteria, members of the genera *Burkholderia*, following by *Ralstonia picketti* and the Bacteroidetes *Flexibacter cf*. *sancti*. There are two major concerns about observing MDR bacteria in mineral water. The first is related to the observation that some of these bacteria are confirmed or suspected nosocomial or opportunistic pathogens; the second concern is related to the possibility of resistance transfer to human pathogens [8]. For instance, some non-fermenting Gram-negative bacilli such as *Ralstonia pickettii*, Bcc and other *Burkholderia spp*. pose a significant problem in the clinical environment, because they are common causes of nosocomial infections [24] and they are also frequently related to pulmonary infections in cystic fibrosis patients [37] [14]. *R*. *pickettii* is emerging as an opportunistic pathogen in both the hospital setting and in environmental sources [24]. Many cases of *R*. *pickettii* infection are caused by contaminated solutions including water for injection and saline solutions made with purified water [25], and these contaminations have led to both bloodstream (bacteremia) and respiratory infections [24]. Resistant infections are becoming more difficult or even impossible to treat with current antibiotics, leading to infections that cause higher morbidity and mortality, and imposing huge costs on our society [38]. The other worry is the existence of resistance determinants that can be transferred to other bacteria. In recent years, increasing resistance involving many common human pathogens has been found, and many of these bacteria and/or their modes of resistance came from the natural environment, including bacteria within soils and water [39] [40]. Moreover, exposure to bacteria from human flora to environmental bacteria with diverse elements of the resistome may accelerate the evolution of resistance by increasing the abundance and distribution of resistance genes within the resistome that can be critical to the development of clinical resistance.

## Conclusions

Two brazilian bottled mineral water tested in this study were confirmed as a source of opportunistic pathogens. Also, antimicrobial resistance phenotypes including resistance to 17 antibiotics, evidence that elements of the resistome could be directly transferred to human opportunistic pathogens or to commensal flora to contribute to a MDR phenotype. The bacteria with the highest MDR indices were members of the genus *Burkholderia*, followed by the species *Ralstonia picketti* and *Flexibacter cf*. *sancti*. Some of these species are important opportunistic pathogens. Because the Bcc has been shown to be transmitted to immunocompromised, we propose that the detection of the Bcc could determine the hygienic quality of bottled mineral waters.
